# Modeling Negative Symptom Domain Neurobiology: Protocol for an Observational, Transdiagnostic, Translational Study

**DOI:** 10.2196/92115

**Published:** 2026-05-18

**Authors:** Dan Cătălin Oprea, Ilinca Untu, Diana-Sabina Vieru, Camelia Dascălu, Helene Speyer, Cristina Dobre, Ohad Green, Romeo Petru Dobrin, Michael Davidson, Jonathan Rabinowitz, Bogdan Ionel Tamba

**Affiliations:** 1 Grigore T. Popa University of Medicine and Pharmacy, Iași, Romania; 2 Advanced Research and Development Center for Experimental Medicine Prof. Ostin C. Mungiu - CEMEX, Grigore T. Popa University of Medicine and Pharmacy, Str. Mihail Kogălniceanu nr. 9–13, Iași, 700454, Romania, 40 232267801; 3 Institute of Psychiatry Socola, Iași, Romania

**Keywords:** negative symptoms, avolition, anhedonia, schizophrenia, bipolar disorder, major depressive disorder, autism, dementia

## Abstract

**Background:**

Negative symptoms (NS) such as anhedonia (reduced pleasure), avolition (reduced motivation), asociality (social withdrawal), blunted affect (diminished emotional expression), and alogia (poverty of speech) are associated with poor functional outcomes in psychiatric and neurological disorders and are an unmet treatment need. Current medication primarily targets positive or affective symptoms, leaving NS neurobiology unaddressed. A critical research gap exists in understanding whether these symptoms share a common biological architecture across different diagnoses or whether they emerge from distinct pathological pathways.

**Objective:**

This observational, single-center study aims to characterize NS across a transdiagnostic sample of individuals with mental disorders and related conditions, with a particular focus on avolition, its biological correlates, and associated neurocognitive and electrophysiological profiles and the aim of determining whether particular NS consistently occupy central positions across disorders or centrality patterns differ by diagnosis. By linking these symptom profiles to underlying neurocognitive, electrophysiological, and genetic markers, the study seeks to disentangle shared versus disorder-specific mechanisms.

**Methods:**

In total, 300 participants with a primary diagnosis of schizophrenia, bipolar disorder, unipolar depressive disorder, autism, or dementia will be recruited, with a target of at least 50 individuals in each diagnostic group. Over a 4-day schedule, participants will undergo clinical (Brief Negative Symptom Scale, Positive and Negative Syndrome Scale, Hamilton Depression Rating Scale, Personal and Social Performance Scale, Udvalg for Kliniske Undersøgelser Side Effect Rating Scale, and Barnes Akathisia Rating Scale), neurocognitive (Brief Assessment of Cognition in Schizophrenia, and Temporal Experience of Pleasure Scale), and biological (resting-state electroencephalography and blood sampling for polygenic risk score) assessments. Network analysis will compute symptom centrality to determine if avolition acts as a transdiagnostic hub. Structural equation modeling will link network profiles to neurophysiological and genetic data. This methodology is designed to identify convergent biological markers, clarify avolition’s role in symptom heterogeneity, and refine understanding of NS as multidimensional phenomena beyond traditional diagnostic boundaries.

**Results:**

The initial version of the study protocol was developed in February 2024. The finalized protocol was completed on May 8, 2024, and updated on January 11, 2025, to incorporate minor methodological clarifications. Participant recruitment and data collection commenced on July 1, 2024, and are ongoing at the time of manuscript submission. By March 2026, the study had enrolled 286 participants. Data quality control and preliminary analyses are performed concurrently with data collection. Final statistical analyses and dissemination of results are planned following completion of the recruitment phase.

**Conclusions:**

This study will provide critical insights into the characterization and underlying mechanisms of NS across psychiatric disorders. By focusing on avolition, reward processing, and their interaction with neurocognitive and social cognitive deficits, it will help identify potential biological and electrophysiological markers of NS. The findings may guide the development of more precise assessment tools and inform novel therapeutic strategies, with broad translational impact for improving outcomes in individuals with serious mental illnesses and related conditions.

## Introduction

### Background

Mental disorders represent a major global health burden, being among the top 10 leading causes of disability [1]. Depression and anxiety are the most prevalent conditions, whereas psychotic disorders such as schizophrenia affect approximately 0.33% to 0.75% of individuals worldwide [2-4].

Negative symptoms (NS) represent a core dimension of psychopathology across the spectrum of serious mental illnesses, including schizophrenia, bipolar disorder, major depressive disorder, and neurocognitive disorders. Unlike positive symptoms, which reflect an excess or distortion of normal function, NS involve a reduction or loss of fundamental psychological processes [5]. Within severe mental illnesses such as schizophrenia, bipolar disorder, and unipolar depressive disorder, as well as in neurocognitive disorders such as dementia, NS remain a significant unmet treatment need [5].

NS refer to a reduction in or loss of normal psychological functions and are classically grouped into two domains: (1) the experiential domain, comprising anhedonia (reduced ability to experience pleasure), avolition (marked reduction in motivation and a diminished ability to initiate, sustain, and complete goal-directed activities), and asociality (reduced interest in social interactions and diminished desire to form or maintain relationships); and (2) the expressive domain, including blunted affect (marked reduction in the expression of emotions, observed as limited facial expressions, reduced eye contact, monotone speech, and decreased use of gestures) and alogia (poverty of speech and reduced verbal output). Importantly, NS are strongly associated with poor functional outcomes, impaired quality of life, and limited response to current therapeutic approaches [5].

In the neo-Kraepelinian era, psychiatric nosology has largely conceptualized mental disorders as natural kinds, understood as discrete disease entities whose observable symptoms are taken to reflect underlying, latent pathological processes. This framework has shaped diagnostic systems by prioritizing reliability; categorical classification; and the assumption that symptoms cohere because they are manifestations of a single, unifying disorder [6]. However, accumulating empirical evidence has increasingly challenged this view. Research has demonstrated substantial symptom overlaps across diagnostic categories, high rates of comorbidity, and heterogeneity within diagnoses, calling into question the notion that traditional categories map neatly onto distinct biological or psychological mechanisms.

In response to the view that mental disorders are discrete entities, more recent approaches have shifted focus from distinct disorders to symptom domains that cut across diagnostic boundaries. Within this transdiagnostic perspective, symptoms such as anhedonia, anxiety, or cognitive dysfunction are investigated as phenomena in their own right, potentially sharing mechanisms across multiple conditions. Building on this shift, network-based models represent a further conceptual departure by rejecting the assumption of a latent disease entity altogether. Instead, they conceptualize psychopathology as emerging from direct, dynamic interactions among symptoms themselves such that symptoms mutually reinforce and sustain one another over time. From this viewpoint, mental disorders are not the causes of symptoms but, rather, the emergent patterns arising from complex symptom networks, with important implications for diagnosis, etiology, and intervention [7].

Recent advances in behavioral, cognitive, and pathophysiological research highlight the need for a more precise characterization of NS to enable the development of innovative treatments. There is evidence suggesting that, at least in part, NS may share common biological substrates and transdiagnostic manifestations. Repeated analyses of neural networks involved in emotional and cognitive processing indicate that avolition is a central component of NS, playing a role in the generation and maintenance of other symptoms. Moreover, improvement in avolition is often accompanied by amelioration of the broader spectrum of NS [8].

Network theory offers a useful framework for advancing the understanding of NS as transdiagnostic phenomena because it shifts the focus from diagnostic categories to the dynamic relations among symptoms. Network models allow these symptoms to be examined as interacting components of a system rather than as passive indicators of an underlying disorder [9]. By modeling symptoms as nodes and their associations as edges, network analysis can test whether NS form stable, coherent clusters across diagnoses or whether their organization, centrality, and connectivity differ among conditions [7]. This approach makes it possible to identify symptoms that act as central hubs or bridges linking motivational, affective, cognitive, and functional domains, thereby clarifying which NS may play a driving vs downstream role in different diagnostic groups [9].

In line with the overarching aim of characterizing NS across diagnostic categories, this study further seeks to examine the relative importance of individual NS within symptom networks by assessing measures of node centrality. Drawing on network theoretical approaches, centrality indexes such as strength, closeness, and betweenness are used to identify symptoms that are most influential within the network of NS. Symptoms with high strength may exert broad influence through strong direct associations with other symptoms, whereas symptoms with high closeness or betweenness may play a key role in maintaining or propagating symptom dynamics across the network. By comparing centrality profiles across diagnostic categories, the study aims to determine whether particular NS consistently occupy central positions across disorders or whether centrality patterns differ by diagnosis. This approach directly supports the study’s goal of disentangling shared vs disorder-specific mechanisms underlying NS and may help identify key targets for transdiagnostic intervention.

### Objectives

The overall aim of this project is to characterize persistent NS in a transdiagnostic sample of individuals with severe mental illnesses by investigating the mechanisms underlying different domains of these symptoms. Specifically, the main objectives of this study are to (1) characterize NS in a transdiagnostic sample by modeling their interrelations using network analysis and compare network structure and symptom centrality across diagnostic categories to distinguish shared vs disorder-specific mechanisms; (2) examine whether avolition occupies a central position within NS networks, supporting its proposed role in generating and maintaining other NS domains; (3) investigate associations between symptom centrality, particularly of avolition, and biological markers, including genetic and other biological measures; (4) examine alterations in reward anticipation and reward experience in relation to NS profiles and motivational deficits and their interactions with neurocognitive and social cognitive dysfunctions; and (5) link network-derived symptom profiles to electrophysiological measures, comparing patterns across symptom profiles, diagnostic categories, and treatment response.

Participants will be recruited according to *International Classification of Diseases, 10th Revision*, criteria from the following diagnostic categories: schizophrenia (all forms), schizoaffective disorder (all forms), persistent delusional disorder (including the schizophrenia spectrum), major depressive disorder, bipolar affective disorder, autism spectrum disorder (diagnosed during childhood or adolescence), and various forms of dementia. An effort will be made to include a similar number of patients in each diagnostic group; however, depending on availability, group sizes may vary. The estimated cohort for this study is 300 patients. To achieve its objectives, the study will use a multimodal assessment protocol including psychometric and clinical scales, neurophysiological recordings, and biological sampling.

## Methods

### Study Design and Setting

This is an observational, single-group, exploratory study. All study visits will be conducted at the Socola Institute of Psychiatry, an academic hospital affiliated with Grigore T. Popa University of Medicine and Pharmacy, Iași, Romania. Data collection will take place exclusively at this site. The trial will recruit an estimated 300 participants across multiple diagnostic categories of severe mental illnesses and related disorders. Participants will be recruited from both inpatient and outpatient treatment settings. Personal data will be anonymized to ensure confidentiality. An effort will be made to include a similar number of patients in each diagnostic group, aiming for at least 50 participants per group, with possible increases up to 80 depending on availability. The study framework is exploratory, aiming to characterize persistent NS, identify potential biomarkers, and investigate underlying mechanisms across diagnostic groups.

### Eligibility Criteria

Participants will be adults aged 18 to 65 years who present with a primary *International Classification of Diseases, 10th Revision*, diagnosis of schizophrenia, bipolar disorder, unipolar depressive disorder, autism, or dementia, with a minimum score of 20 on the NS subscale of the Positive and Negative Syndrome Scale (PANSS) [10]. Participants unable to speak Romanian, who are simultaneously enrolled in other clinical trials, or who have somatic comorbidities that may influence study outcomes will be excluded.

### Sample Size

For this exploratory transdiagnostic study, we plan to recruit 300 participants distributed across 5 diagnostic groups (approximately 60 per group). This sample size is sufficient to detect small to medium between-group effects on continuous outcomes (eg, NS domain scores and reward and electrophysiological measures) using one-way or factorial ANOVA or linear models with an α of .05 and power of at least 0.80 [11]. It also provides adequate precision for multivariable regression and structural equation models examining the role of avolition and biological markers, maintaining a conservative ratio of at least 10 to 15 participants per estimated parameter [12,13]. Given the primary focus on characterizing patterns and mechanisms rather than testing a single primary contrast, the chosen sample balances statistical power, model stability, and feasibility across the planned clinical, cognitive, biological, and electrophysiological analyses [14].

### Ethical Considerations

The study protocol and all associated procedures were reviewed and approved by the institutional review board of the Grigore T. Popa University of Medicine and Pharmacy, Iași, Romania (approval 760253). The study will be conducted in accordance with the Declaration of Helsinki and local ethical guidelines for human subject research. Participation in the study will not alter the patients’ ongoing clinical management.

Written informed consent will be obtained from all participants prior to their enrollment and before any study-specific procedures (including clinical interviews, electroencephalography [EEG] recordings, or blood draws) are conducted. In cases involving participants with severe cognitive impairment, written informed consent will be obtained from their legally authorized representative or guardian in accordance with local legal and ethical standards.

Strict measures will be implemented to safeguard participant information. All clinical, biological, and neurophysiological data will be deidentified immediately upon collection. Each participant will be assigned a unique numeric study ID, which will be used on all subsequent data files, blood samples, and EEG recordings. The key linking the study ID to personal identifying information will be stored separately on secure, encrypted servers with access limited to core study personnel. Audio and video recordings of Brief Negative Symptom Scale (BNSS) sessions will be similarly encrypted. Hard copies of documents, including signed consent forms, will be securely archived in locked cabinets at the CEMEX research infrastructure.

Participants will not receive any financial compensation for taking part in this study.

### Data Collection

All data will be collected by trained study coordinators and clinical staff at the Socola Institute of Psychiatry in compliance with institutional and ethical regulations for patient privacy and data security. Standardized clinical scales, neurophysiological assessments, and biological samples will be obtained according to the study visit schedule to ensure consistency and reproducibility.

NS will be assessed using the BNSS [15], a 13-item semistructured clinical interview evaluating the 5 consensus domains of NS (anhedonia, asociality, avolition, blunted affect, and alogia) administered by trained raters at multiple visits. To enhance reliability, interviews will be audio and video recorded, capturing both patient responses and examiner behavior. These recordings will be stored on secure, encrypted servers and used for quality monitoring and interrater reliability checks. To ensure a comprehensive clinical, functional, and neurocognitive characterization, several additional validated instruments will be administered. Overall psychopathology will be assessed using the PANSS [10], whereas the Hamilton Depression Rating Scale [16] will evaluate depressive symptom severity to differentiate primary NS from depressive phenomena. Psychosocial functioning will be measured via the clinician-rated Personal and Social Performance Scale [15,16]. To account for secondary NS induced by medications, side effects will be systematically monitored using the Udvalg for Kliniske Undersøgelser Side Effect Rating Scale [13] and the Barnes Akathisia Rating Scale [14]. Finally, cognitive, motivational, and reward processing domains will be evaluated using the Brief Assessment of Cognition in Schizophrenia [17] for global neurocognitive functioning, the Temporal Experience of Pleasure Scale [18] to differentiate anticipatory from consummatory anhedonia, and the Defeatist Performance Beliefs Scale [19] to capture negative expectancy appraisals conceptually linked to avolition.

EEG recordings will be conducted by a certified technician during hospitalization following a standardized protocol. Each session will include an 8-minute resting-state acquisition divided into alternating 2-minute blocks of eyes open and eyes closed. Data will be processed for event-related potentials and spectral power analyses aggregated at the group and symptom profile levels. Raw EEG data will be securely transferred to the CEMEX research infrastructure for storage and advanced processing.

Venous blood samples (3 mL; ethylenediaminetetraacetic acid tubes) will be collected at the final visit. Samples will be kept at 4 °C for a maximum of 4 hours prior to laboratory transfer. Following centrifugation, aliquots of 500 μL to 1 mL will be stored at –80 °C. Genomic DNA will be isolated and quality controlled using NanoDrop (Thermo Fisher), Qubit (Thermo Fisher), and agarose gel electrophoresis. Genotyping will be performed via genome-wide microarray or whole-genome sequencing, and polygenic risk scores (PRSs) for persistent NS will be calculated. All biological samples will be processed and stored at CEMEX. A detailed timeline of participant enrollment and the schedule of all clinical, cognitive, and biological assessments are summarized in Table 1.

**Table 1.** Timeline of enrollment and assessments.

	Study period			
	T–1	T0	T1 (day 2 after enrollment)	T2
Enrollment				
Project description	✓			
Q&A^a^	✓→	→	→	→✓
General clinical evaluation	✓			
Eligibility screen	✓			
Informed consent		✓		
Assessments				
PANSS^b^		✓		
BNSS^c^		✓→	→✓	
HAM-D^d^			✓	
UKU^e^				✓
BARS^f^				✓
PSP^g^				✓
TEPS^h^				✓
BACS^i^				✓
EEG^j^			✓	
Blood draw				✓

a Q&A: questions and answers.

b PANSS: Positive and Negative Syndrome Scale.

c BNSS: Brief Negative Symptom Scale.

d HAM-D: Hamilton Depression Rating Scale.

e UKU: Udvalg for Kliniske Undersøgelser Side Effect Rating Scale.

f BARS: Barnes Akathisia Rating Scale.

g PSP: Personal and Social Performance Scale.

h TEPS: Temporal Experience of Pleasure Scale.

i BACS: Brief Assessment of Cognition in Schizophrenia.

j EEG: electroencephalography.

### Data Security Protocols

All clinical, biological, and neurophysiological data will be deidentified and stored on encrypted servers with restricted access. Consent forms and paper records will be securely archived at CEMEX. Duplicate data entry and regular audits will ensure integrity and completeness. Audio and video recordings of BNSS sessions will provide an additional layer of quality control and facilitate interrater calibration.

All study staff will undergo structured training and certification on the administration of scales and procedures prior to participant contact.

### Statistical Analysis

The statistical analysis plan focuses on characterizing NS across a transdiagnostic cohort with prominent NS. Analyses will begin with descriptive and multivariate methods to map the structure, severity, and distribution of NS domains followed by regression-based and structural equation approaches to examine the central role of avolition in shaping and maintaining other NS. Biological correlates of avolition will be explored through both targeted and data-driven analyses of peripheral biomarkers, neuroimaging indexes, and electrophysiological measures. Additional models will investigate the pathophysiological mechanisms underlying distinct NS domains, integrating behavioral, cognitive, and biological data. Reward processing, both anticipation and consummation, will be examined in relation to motivational deficits and their interaction with neurocognitive and social cognitive performance. Psychological networks of the interplay of symptoms will be examined as per network theory analysis methods [18,19]. Electrophysiological profiles will be compared across symptom profiles, diagnostic groups, and treatment response categories, with predictive models assessing whether baseline neural signatures forecast clinical outcomes. Across all analyses, models will adjust for relevant covariates, apply appropriate corrections for multiple testing, and use robust methods to address missing data.

All analyses will be conducted using R (eg, the *qgraph*, *bootnet*, *psychonetrics*, or *lavaan* packages; R Foundation for Statistical Computing) and complementary EEG and genetic analysis packages. Statistical significance will be evaluated using 2-sided tests with an α value of .05. For families of related comparisons, the false discovery rate (FDR) will be controlled using the Benjamini-Hochberg procedure. Effect sizes (eg, standardized coefficients, Cohen *d*, and Hedges *g*) will be reported alongside CIs.

### Data Preparation

Descriptive statistics will be computed for all clinical, cognitive, EEG, and genetic variables. Distributions will be inspected for skewness, kurtosis, and outliers (>3.5 SD). Item-level missingness of 10% or less within a scale will be imputed using person-mean substitution; otherwise, the scale score will be treated as missing. Multivariate models will use full information maximum likelihood or multiple imputation under a missing at random assumption. Variables with substantial nonnormality will be transformed as appropriate.

### Objective 1: Network Structure and Transdiagnostic vs Disorder-Specific Mechanisms

#### Network Estimation

NS networks will be estimated using Gaussian graphical models based on polychoric correlations among BNSS items. Networks will be regularized using the graphical least absolute shrinkage and selection operator with extended Bayesian information criterion model selection (γ=0.5). Primary analyses will use item-level BNSS nodes; secondary analyses will use domain-level nodes and expanded networks incorporating PANSS negative items.

Networks will be estimated (1) in the full transdiagnostic sample and (2) separately within each diagnostic group (schizophrenia, bipolar disorder, unipolar depression, autism, and dementia) provided that sample size supports stable estimation.

#### Centrality and Stability

Centrality indexes (strength and expected influence) will be computed for each node. Stability will be evaluated using nonparametric bootstrapping and case-dropping procedures, with centrality stability coefficients of 0.25 or higher considered acceptable and coefficients of 0.50 or higher preferred. Bootstrapped CIs will be generated for edge weights and centrality estimates.

#### Network Comparisons

Between-group differences in network structure will be tested using the network comparison test. Tests will assess (1) global strength invariance, (2) network structure invariance, and (3) node-specific centrality differences. FDR correction will be applied across multiple comparisons. Convergent patterns across groups will be interpreted as transdiagnostic mechanisms, whereas significant divergences will be considered disorder specific.

### Objective 2: Centrality of Avolition

Avolition will be examined for relative centrality within each network. Hypotheses are that avolition will (1) exhibit higher strength centrality than the mean of all other nodes in the transdiagnostic network and (2) rank among the most central nodes across diagnostic groups.

Bootstrapped difference tests will compare avolition centrality to other nodes and domains. Sensitivity analyses will include domain-level networks, alternative regularization parameters, and networks excluding items with overlapping content.

### Objective 3: Associations Between Symptom Centrality and Biological Markers

#### Individual-Level Network Metrics

To link group-level network structure to individual biology, participants will receive centrality-weighted NS scores derived by weighting BNSS item scores by group-level centrality indexes. BNSS domain scores (including avolition) will also be used as proxies for domain-specific severity.

#### Genetic and Biological Associations

Linear regression models will examine associations between PRSs (schizophrenia, bipolar disorder, depression, and cognition-related traits) and (1) avolition severity and (2) centrality-weighted NS scores. Models will adjust for age, sex, diagnostic group, medication status, and ancestry principal components.

Multivariate models and structural equation modeling (SEM) will evaluate whether PRSs differentially predict specific NS domains and whether biological markers (eg, EEG spectral power or connectivity) mediate or moderate these associations.

### Objective 4: Reward Processing, NS, and Cognitive Dysfunction

#### Symptom Profile Identification

Latent profile analysis will be conducted using BNSS domain scores to identify NS subgroups. Model selection will be guided by the Bayesian information criterion, the Akaike information criterion, entropy, and interpretability.

#### Reward and Cognitive Associations

Reward anticipation and consummatory experience (Temporal Experience of Pleasure Scale) will be compared across symptom profiles using analyses of covariance adjusting for demographic and clinical covariates. Interactions between avolition and neurocognitive performance (Brief Assessment of Cognition in Schizophrenia) or social cognition (if available) will be tested to evaluate whether cognitive deficits amplify motivational impairments.

#### Integrated SEM

SEM will model relationships among NS domains, reward processing, cognition, and functional outcome (Personal and Social Performance Scale). Competing models will test whether avolition exerts direct effects on reward deficits and whether these, in turn, predict functional impairment.

### Objective 5: Electrophysiological Correlates of Symptom Profiles

#### EEG Feature Extraction

Resting-state EEG will undergo standard preprocessing (filtering, artifact correction, and rereferencing). Spectral power (delta, theta, alpha, beta, and gamma waves) and connectivity metrics (eg, coherence and phase locking) will be extracted from predefined regions of interest.

#### EEG–Symptom Profile Associations

EEG features will be compared using the methodologies presented by Dang et al [17] across network-derived symptom profiles and diagnostic groups using multivariate ANOVA or multivariate analysis of covariance adjusting for age, sex, medication, and PRS. Post hoc tests will be FDR corrected. Exploratory analyses will examine whether EEG patterns associated with avolition or centrality-weighted symptom scores differ across diagnostic categories.

## Results

The initial version of the study protocol was developed in February 2024 and formally approved on May 8, 2024, with an updated version finalized on January 11, 2025. Participant recruitment and data collection began on July 1, 2024, at the Socola Institute of Psychiatry and are currently ongoing. The results of this study are expected to be published in October 2026.

By March 2026, the study had enrolled a total of 286 participants across the targeted diagnostic categories. No deviations from the originally approved protocol have occurred.

Data collection is being conducted on a rolling basis during inpatient admissions. Interim data cleaning and quality control procedures are performed continuously throughout the recruitment period. Final data cleaning, statistical analyses, and interpretation of results will be conducted following completion of enrollment, with manuscript preparation planned thereafter.

## Discussion

### Anticipated Findings

Despite the impact of NS on functional recovery, long-term disability, and quality of life, their underlying mechanisms remain only partially understood, particularly when considered across diagnostic boundaries rather than within schizophrenia alone. NS—especially avolition—are increasingly recognized as multidimensional phenomena linked not only to motivational and reward processing deficits but also to broader disturbances in cognitive control, effort valuation, and neurobiological systems governing goal-directed behavior [9,20]. Importantly, persistent NS have been identified in diverse clinical populations, including mood disorders, schizoaffective disorder, and individuals at high clinical risk, highlighting the need for a transdiagnostic approach to understanding their etiology and course [21].

This observational study seeks to address this gap by systematically characterizing the clinical, neurocognitive, electrophysiological, and biological correlates of NS, with a particular emphasis on avolition, in a transdiagnostic sample of individuals with severe mental illnesses and related conditions. Prior work suggests that motivational impairments may arise from disruptions in reward anticipation, aberrant salience processing, impairments in executive function, and altered dopaminergic and inflammatory signaling [22]. By integrating standardized psychometric assessments with neurofunctional measures—such as EEG indexes of reward responsiveness—and polygenic risk profiles, this study aims to clarify how these diverse mechanisms interact to produce clinically significant motivational deficits.

Such multimodal characterization is essential as emerging evidence indicates that NS may reflect convergent disturbances across neurobiological systems rather than a single pathway. For example, electrophysiological markers such as reduced reward positivity and attenuated error-related negativity have been linked to amotivation across diagnostic groups [23], whereas polygenic risk for schizophrenia and depression has been associated with deficits in reward processing and goal-directed behavior even in nonclinical populations [24]. By examining these domains concurrently, this study contributes to a more integrated model of NS pathophysiology.

Ultimately, findings from this research are expected to support the refinement of diagnostic frameworks that better capture motivational dysfunction across psychiatric conditions. Moreover, elucidating the neurocognitive and biological underpinnings of avolition may inform the development of targeted, mechanism-based interventions—such as cognitive motivational remediation, reward-based behavioral therapies, or biomarker-guided pharmacological strategies—aimed at improving functional outcomes for individuals with severe mental illness [25,26].

NS represent a major and persistent challenge across the spectrum of severe mental illnesses, contributing substantially to functional disability, poor quality of life, and limited response to current treatments. Despite their clinical relevance, NS remain insufficiently characterized from a transdiagnostic and biologically informed perspective. By systematically assessing NS across multiple diagnostic categories, this study is expected to provide a more refined understanding of their phenomenology beyond traditional disorder-based frameworks.

Therefore, clarifying the role of avolition may help explain the heterogeneity of NS presentations across diagnostic groups. By integrating standardized psychometric assessments with electrophysiological measures and polygenic risk profiling, this study is expected to identify convergent biological and neurofunctional patterns associated with NS severity.

The transdiagnostic nature of the sample represents a key strength of this investigation. Rather than focusing exclusively on schizophrenia, this study includes individuals with affective disorders and other conditions characterized by NS, thereby addressing an important gap in the literature.

Overall, the anticipated findings are expected to contribute to a more nuanced understanding of NS as biologically grounded, multidimensional phenomena.

### Limitations

This study has several limitations that should be acknowledged. First, participants will be recruited from a single academic psychiatric center, which may limit the generalizability of the findings. Although the transdiagnostic nature of the sample enhances conceptual relevance, local clinical practices, patient characteristics, and referral patterns at the Socola Institute of Psychiatry may not fully represent other settings or populations. Multicenter studies will be required to confirm the robustness of the observed associations across different clinical contexts. Second, the assessment of NS will rely partly on clinician-rated and self-report instruments. While validated scales and trained raters will be used, self-report measures remain susceptible to recall bias, limited insights, and response variability, particularly in patients with cognitive impairment or severe psychopathology. In addition, symptom severity may fluctuate during hospitalization due to changes in clinical state or ongoing pharmacological treatment, potentially influencing assessment stability across visits. Third, despite standardized training procedures, the involvement of multiple raters introduces the possibility of interrater variability. Finally, participant attrition or exclusion during the assessment process may occur due to symptom instability, limited cooperation, or mistrust related to psychotic symptoms, which could introduce selection bias. These limitations should be considered when interpreting the findings. Nevertheless, the comprehensive multimodal approach and transdiagnostic focus of this study provide a strong foundation for future longitudinal and multicenter research aimed at refining the biological and clinical characterization of NS.

### Conclusions

This study will provide critical insights into the characterization and underlying mechanisms of NS across psychiatric disorders. Focusing on avolition, reward processing, and their interaction with neurocognitive and social cognitive deficits will help identify potential biological and electrophysiological markers of NS. The findings may guide the development of more precise assessment tools and inform novel therapeutic strategies, with broad translational impact for improving outcomes in individuals with severe mental illnesses and related conditions.

## Supplementary material

10.2196/92115 Peer Review Report 1 Peer review report from the National Recovery and Resilience Plan (PNRR), Pylon III, Section I8. Development of a Program to Attract Highly Specialised Human Resources from Abroad in Research, Development and Innovation Activities (Ministry of Research, Innovation and Digitization, Government of Romania).

## Abbreviations

BNSS: Brief Negative Symptom Scale
EEG: electroencephalography
FDR: false discovery rate
NS: negative symptoms
PANSS: Positive and Negative Syndrome Scale
PRS: polygenic risk score
SEM: structural equation modeling
