# Supplementary material for: Modeling Negative Symptom Domain Neurobiology: Protocol for an Observational, Transdiagnostic, Translational Study
Source: JMIR Res Protoc. 2026 May 18;15:e92115. doi: 10.2196/92115 (PMC13183023; doi:10.2196/92115)
Supplement: Peer Review Report 1 [file resprot-v15-e92115-s001.pdf]

Consolidated Evaluation Report

**REGISTRATION CODE UEFISCDI**	F-PNRR-I8-2022-0430
**REGISTRATION CODE MIPE**	997004516
**PROJECT TITLE**	Modelling negative symptom domains neurobiology: a transdiagnostic, translational study
**Main Domain**	LS5_12 - Mental disorders
**Secondary Domain**	LS5_18 - Innovative methods and tools for neuroscience
**Secondary Domain**	LS7_1 - Medical imaging for prevention, diagnosis and monitoring of diseases

1. Principal Investigator (PI) - 40% of the total score

Criterion 1.1
Quality of the PI's research output - 40% of the total score of Criterion 1

Score: 4.00
12,80 point(s)

Evaluate to what extend the PI's research has led to progress in their field of expertise, in general (i.e., not only in the narrow field/theme of the project). Comment on the importance of the PI's scientific discoveries, as reflected in their track record or other achievements.

Strengths	Weaknesses
- Very productive researchers in terms of volume of research output, especially scholarly publications.<br>- The Reviewers also noted the co-authorship of a patent.<br>- Impact of publications in terms of citations is noted are relatively strong.	*- The Reviewers reported that the applicant is not listed as Principal Investigator of research grants ; all grants reported mention the applicant as co-investigator.*

Criterion 1.2
Visibility and impact of the PI's research output - 30% of the total score of Criterion 1

Score: 3.50
8,40 point(s)

Evaluate to what extent the PI's scientific output is internationally recognized. Comment on the international visibility of the PI's scientific output as reflected, for example, in attract research funding, in citations in top journals, number of citations, relevance of their published work (For 1.1.-1.2. please take into account the scientific output in relation to the current career stage of the PI).

Strengths	Weaknesses
- Participation in multiple consortia and networks as a co-investigator.<br>- As mentioned above, the relatively large number of scholarly publications have been well cited so far.	*- The geographic concentration of research projects to which the applicant has participated so far seemed limited to the Reviewers.<br>- They also noted the lack of experience in coordinating and leading large, complex research projects as Principal Investigator.<br>- Although the scholarly production is large in volume, the applicant mostly appears a a "middle co-author", not as a lead or senior author. Therefore, the significance of their contributions is difficult to assess.*

Criterion 1.3
PI's ability to tackle the proposed topic - 30% of the total score of Criterion 1

Score:
4.00
9,60
point(s)

Evaluate to what extend the PI's research output is relevant for the present project. Comment on how the previously published work or previous projects of the PI relates to the proposed research.

Strengths	Weaknesses
- The Reviewers recognized the clinical expertise of the applicant, which is an asset in the proposed field of research.	*- The Reviewers emphasized that the scope of the proposed project is very broad and questioned the capacity (in time and expertise) of the applicant to lead and coordinate its execution, especially in terms of large project management skills, which are not documented in the application or the applicant's track record so far.<br>- Time management will also be a challenge because of the PIs commitments to the clinic and other research projects.<br>- It is unclear how the preclinical aspects of the proposed research will be managed by the PI because of their lack of expertise in that area.*

2. Research Project - 60% of the total score

Criterion 2.1
State-of-the-art and originality/innovation - 30% of the total score of Criterion 2

Score:
3.75
13,50
point(s)

Evaluate whether the problem addressed by the project is clearly identified in relation to the state-of-the-art in the field. Comment on the originality and novelty of the proposed solution. If previous projects of the applicant addressing a similar topic are mentioned, comment on the novel aspects investigated in the present project.

Strengths	Weaknesses
- The research area and questions were perceived as timely and relatively significant.<br>- The proposal is clearly written, with sufficient background and motivation.	*- The novelty of the proposed research was perceived as either unclear or relatively limited by the Reviewers.<br>- The sample sizes proposed were also judged as too small to demonstrate the expected effects, especially in a group of participants where high variability is expected.*

Criterion 2.2
Research objectives, methodology and work plan - 30% of the total score of Criterion 2

Score:
3.75
13,50
point(s)

Evaluate the clarity and coherence of the scientific objectives. To what extent is the proposed methodological approach suitable for reaching these objectives? How effective is the work plan (timelines, milestones, deliverables) in terms of achieving the proposed objectives? Comment on the coherence of the approach in terms of activities and time scales.

Strengths	Weaknesses
- The overall research objectives were appreciated by the Reviewers, with appropriate methodology and approaches.	*- The Reviewers found that the actual work packages and deliverables were not described with sufficient details to properly assess their significance and expected outcomes.<br>- They raised the question of how the vulnerable patients recruited will be enrolled in the study. More details concerning the ethical aspects of the research conduct need to be provided.*

Criterion 2.3
Feasibility (resources, research team and preliminary results) - 20% of the total score of Criterion 2

Score: 3.75 9,00 point(s)

To what extent is the success of the project plausible? To what extent the human and material resources available for the project will ensure successful implementation of the project? Are there any preliminary results presented in support of the hypothesis and proposed solution?

Strengths	Weaknesses
- The Reviewers appreciated the strengths of the clinical environments and the access to research participants.	*- The Reviewers found that on several aspects of the project, the experience and expertise of CEMEX would need to be better explained to strengthen the feasibility of the proposed resaerch.<br>- Previous collaborations with the proposed partners of the project, e.g., through pilot studies, would be a strength and bolster the feasibility of the proposed research.<br>- Again, the lack of management experience of the applicant with large-scale research projects is seen as a limiting factor in the execution of the research plan.<br>- Another limiting aspect is the apparent limited experience of the neuroimaging team: this would need to be better explained and documented.*

Criterion 2.4
Risks and contingency plans - 10% of the total score of Criterion 2

Score: 2.50 3,00 point(s)

To what extent the risk analysis correctly identifies potential pitfalls? Also comment on the effectiveness of the alternative solutions proposed.

Strengths	Weaknesses
- Some risks are anticipated and mitigation strategies are mentioned.	*- The Reviewers found that most mitigation measures are not on par with the expected standards of this type of research.<br>- Major risks in terms of participant recruitment and retention need to be anticipated and mitigated with realistic implementation strategies.*

Criterion 2.5
Expected impact and dissemination plan - 10% of the total score of Criterion 2

Score: 3.50 4,20 point(s)

To what extent is the expected scientific output of the proposed work realistically described and how likely is it to lead to significant progress in the field? How will the proposed research impact (the visibility of) the host institution, PI and research team? Also, comment on the quality of the proposed measures to disseminate the scientific output of the proposal. Social, economic, or cultural impact should be considered only if relevant for the proposed research.

Strengths	Weaknesses
- A relatively standard academic dissemination plan is provided.	*- The Reviewers noted several missed opportunities to detail plans and strategies concerning the translational aspects of the research outcomes, in terms of information to the general and patient population, policy makers and relevant clinical teams.*

3. Budget (this section will not be scored)

Criterion 3.1
Please provide an overall assessment of the research budget requested and evaluate to what extent it is justified by the proposed research activities. There will be no score associated with this criterion, but the assessment will be useful to the funding agency in negotiating the final financial award

Overall, the budget was seen as adequate, although the IT components would need to be better justified.	

Total Score
74,00

4	92	Exploring bacterial protein-protein interactions for antibiotic research - BACPROBIO	UNIVERSITATEA BABES BOLYAI	90,7	a îndeplinit pragurile minime impuse
5	162	Adaptive laboratory evolution strategies to increase the lipid accumulation process in oleaginous microorganisms and microbial oil production using agroindustrial residues as substrates.	UNIVERSITATEA BUCURESTI	88,2	a îndeplinit pragurile minime impuse
6	12	Role of HIV-1 matrix protein p17 variants in lymphomagenesis: development of new diagnostic tools and therapeutic approaches	UNIVERSITATEA DE MEDICINA, FARMACIE, STIINTE SI TEHNOLOGIE ”GEORGE EMIL PALADE” DIN TARGU MURES	87,7	a îndeplinit pragurile minime impuse
7	46	Modelling negative symptom domains neurobiology: a transdiagnostic, translational study	UNIVERSITATEA DE MEDICINA SI FARMACIE "GRIGORE T. POPA" DIN IAȘI	87,5	a îndeplinit pragurile minime impuse
		Creating a Research Group of [illegible]			
